# Inflammation: a putative link between phosphate metabolism and cardiovascular disease

**DOI:** 10.1042/CS20190895

**Published:** 2021-01-08

**Authors:** Jakob Voelkl, Daniela Egli-Spichtig, Ioana Alesutan, Carsten A. Wagner

**Affiliations:** 1 Institute for Physiology and Pathophysiology, Johannes Kepler University Linz, Altenberger Strasse 69, Linz 4040, Austria; 2 DZHK (German Centre for Cardiovascular Research), Partner Site Berlin, Berlin 13347, Germany; 3 Department of Nephrology and Medical Intensive Care, Charité-Universitätsmedizin Berlin, Campus Virchow-Klinikum, Augustenburger Platz 1, Berlin 13353, Germany; 4 National Centre of Competence in Research NCCR Kidney.CH, Switzerland and Institute of Physiology, University of Zurich, Winterthurerstrasse 190, Zurich CH-8057, Switzerland

**Keywords:** cardiovascular disease, chronic kidney disease, FGF23, inflammation, phosphate, vascular calcification

## Abstract

Dietary habits in the western world lead to increasing phosphate intake. Under physiological conditions, extraosseous precipitation of phosphate with calcium is prevented by a mineral buffering system composed of calcification inhibitors and tight control of serum phosphate levels. The coordinated hormonal regulation of serum phosphate involves fibroblast growth factor 23 (FGF23), αKlotho, parathyroid hormone (PTH) and calcitriol. A severe derangement of phosphate homeostasis is observed in patients with chronic kidney disease (CKD), a patient collective with extremely high risk of cardiovascular morbidity and mortality. Higher phosphate levels in serum have been associated with increased risk for cardiovascular disease (CVD) in CKD patients, but also in the general population. The causal connections between phosphate and CVD are currently incompletely understood. An assumed link between phosphate and cardiovascular risk is the development of medial vascular calcification, a process actively promoted and regulated by a complex mechanistic interplay involving activation of pro-inflammatory signalling. Emerging evidence indicates a link between disturbances in phosphate homeostasis and inflammation. The present review focuses on critical interactions of phosphate homeostasis, inflammation, vascular calcification and CVD. Especially, pro-inflammatory responses mediating hyperphosphatemia-related development of vascular calcification as well as FGF23 as a critical factor in the interplay between inflammation and cardiovascular alterations, beyond its phosphaturic effects, are addressed.

## Control of inorganic phosphate and calcium balance

### The triangle of intestine–bone–kidney controls systemic inorganic phosphate balance

**Figure 1:**
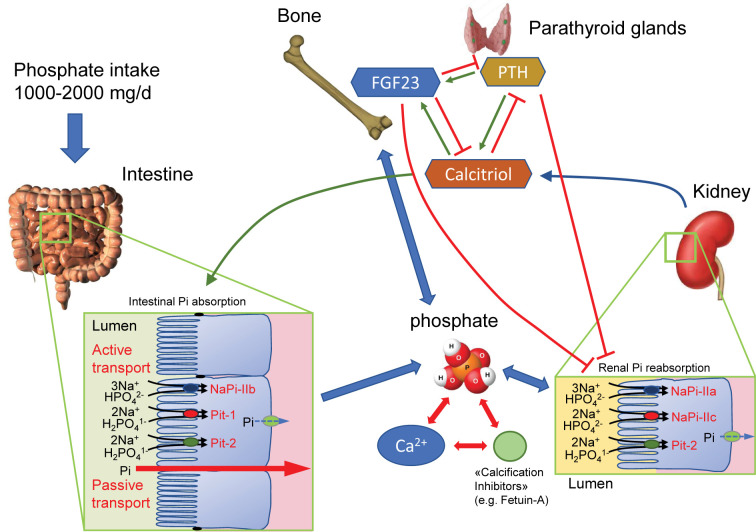
Interplay and control of Pi absorption, handling and excretion Pi is taken up from diet by multiple active and passive transport systems located in the small intestine. In rodents, NaPi-IIb is the main active intestinal Pi transporter. Blood Pi is undergoing constant renal filtration and reabsorption mediated by the NaPi-IIa, NaPi-IIc and PiT-2 Pi transporters. Pi homeostasis is regulated by three major hormones, by the bone-derived FGF23, by PTH and by calcitriol. These hormones are linked by positive (green arrows) and negative (red arrows) feedback loops. Calcitriol, stimulates intestinal Pi absorption, whereas PTH and FGF23 block renal reabsorption. Pi in the extracellular space can interact with calcium to form calciprotein particles (CPPs), a process modulated by calcification inhibitors. Abbreviations: FGF23, fibroblast growth factor 23; PTH, parathyroid hormone.

Inorganic phosphate (Pi) is critically required for a myriad of (metabolic) pathways including synthesis of DNA and RNAs, formation of phospholipids and membranes, generation of ATP/GTP/UTP, cellular signaling by phosphorylation and dephosphorylation, buffering of pH in intracellular fluids and urine, and importantly for stability of bone in the form of hydroxyapatite. On the other side and as discussed in detail in this review, excess Pi can be detrimental and exert toxic effects. Thus, systemic Pi levels are tightly controlled through the actions of several organ system and endocrine networks (Figure 1).

Dietary Pi is absorbed along the small intestine [1]. Pi present in animal protein is easily absorbed after cleavage by alkaline phosphatases present in the brush border membrane of enterocytes lining the small intestine. Even more, Pi salts used by food industry in processed food items are readily absorbed and are often present in large quantities (see below). Organic phosphate is present in grains and plant proteins, but under the form of phytic acid or phytate salts for which the human body does not produce enzymes (phytases) to cleave. Nevertheless, there is low phytase activity present in the human intestine stemming from yeast and bacteria of the gut microbiome. Pi is absorbed in the small intestine either through active transcellular transport or via the passive paracellular route. Active Pi transport is mediated mostly by the Na^+^-dependent Pi transporter NaPi-IIb (SLC34A2), at least in rodents [1,2]. The role of two additional Pi transporters, PiT1 and PiT2 is currently less clear [3,4]. This active transport has a high affinity, but low capacity. It is highly regulated and adapts to dietary Pi intake and systemic Pi levels. In contrast, paracellular Pi transport is mostly driven by the Pi gradient between diet/lumen and blood, i.e. fluxes are high when diet is rich in Pi [5]. It is currently unclear whether paracellular Pi fluxes are regulated and which molecules mediate Pi permeability.

Pi absorbed distributes in the extracellular fluid volume and is in constant exchange with a large intracellular Pi pool in soft tissue, mostly skeletal muscle, and a much larger Pi pool in bone. Pi present in blood represents only approximately 1% of total body Pi. This Pi pool is constantly filtered by the kidney and partly reabsorbed along the nephron. Under a normal dietary Pi content, approximately 80–90% of filtered Pi is reabsorbed. The kidneys play a central role in the control of systemic Pi balance as reabsorption of Pi is highly regulated in contrast with intestinal Pi absorption, which is much less controlled. Renal reabsorption of Pi takes place mostly in the proximal tubule mediated by at least three Na^+^-dependent Pi transporters NaPi-IIa (SLC34A1), NaPi-IIc (SLC34A3) and PiT2 (SLC20A2) located in the brush border membrane [6]. The importance of NaPi-IIa and NaPi-IIc for renal Pi handling has been demonstrated through human inherited disorders affecting these two genes [7]. The expression and activity of NaPi-IIa and NaPi-IIc are highly regulated by a variety of factors including high dietary Pi intake, parathyroid hormone (PTH), fibroblast growth factor 23 (FGF23), dopamine, glucocorticoids, or acidosis, all leading to the down-regulation of transporters and higher urinary Pi excretion. Conversely, growth hormone and insulin-like growth factor 1 (IGF-1), calcitriol, or alkalosis stimulate renal Pi transporters and reduce urinary Pi excretion [6].

### An endocrine triangle regulates Pi homeostasis

As indicated above, several factors and hormones modulate renal Pi handling. Among those, calcitriol, PTH, and FGF23 have gained most attention as very important regulators of Pi homeostasis [8]. These three hormones regulate each other thereby forming a regulatory triangle. An increase in dietary Pi intake or plasma Pi levels stimulates PTH secretion, which enhances bone FGF23 formation and release as well as synthesis of calcitriol by kidneys. FGF23 suppresses PTH and calcitriol levels, whereas calcitriol stimulates FGF23 release and inhibits PTH synthesis and secretion. In addition to these positive and negative feedback loops, all three hormones are regulated by many other factors. Particularly, FGF23 has gained much attention and stimulation of FGF23 production and secretion by interleukin (IL)-1β (IL-1β), IL-6, tumor necrosis factor (TNF), erythropoietin, leptin, iron as well as αKlotho was shown [9]. In contrast, adiponectin, insulin and growth hormone, and IGF-1 suppress FGF23 secretion from bone. αKlotho plays an important role not only as a regulator of FGF23 synthesis, but as an obligatory cofactor binding intact FGF23 and allowing its signaling via the FGF receptor 1c (FGFR1c) [10]. In the absence of αKlotho, FGF23 signals through the FGF receptor 4, but at much higher concentrations that are only found in pathological states such as in chronic kidney disease (CKD) or systemic inflammation [11].

This triangle becomes highly dysregulated in various disease states, most markedly in patients with CKD. FGF23 rises already within hours after an acute kidney injury (AKI), but also during the development and progression of CKD. FGF23 rises very early paralleled by a fall in αKlotho levels [8,12,13]. As kidney function further declines, calcitriol levels fall. Only in later stages of CKD, PTH increases and finally also plasma Pi levels are elevated. The trigger for higher FGF23 may be the increase in pro-inflammatory cytokines such as TNF and IL-6, which are elevated in AKI and CKD (see below). While highly elevated FGF23 levels in patients with CKD are associated with faster progression to the requirement for kidney replacement therapy, cardiovascular morbidity and mortality (see below), neutralization of FGF23 in rodent models of CKD has shown detrimental effects causing hyperphosphatemia with excessive vascular calcification and early mortality [14]. Thus, the early increase in FGF23 together with the later increase in PTH may support renal clearance of Pi in face of lower renal function and thereby maintains normophosphatemia until renal function further declines.

### Dietary Pi provides a metabolic acid load

As discussed below, diets in industrialized countries are rich in inorganic Pi either stemming from animal protein or from Pi salts added to processed food. Both forms of Pi provide not only a Pi load to the body, but also an acid challenge [15,16]. This acid challenge is particularly strong when Pi is released from animal protein, which is also rich in sulfuric amino acids and when metabolized release sulfuric acid that is an obligate acid and must be first buffered and later excreted by the kidneys. Even though Pi serves also as a urinary buffer as the major part of the so-called titratable acidity, Pi salts often add more acid to the body than they help to remove by renal excretion. Thus, when discussing detrimental effects of dietary Pi, the type and source of Pi should always be considered and effects on acid–base balance taken into account. As an example, the detrimental effects of Pi-rich diets on bone involve likely also a component of acid-induced bone dissolution and osteomalacia.

### Systemic calcium metabolism

Calcium also fulfills many essential functions in the human body, most notably as counterion to Pi in forming calcium hydroxyapatite in bone, but also in cellular signaling, as cofactor for many enzymatic reactions or as a regulator of ion channel function and cellular excitability. Even though only approximately 1% of total body calcium is present in the extracellular space and approximately 40% of this extracellular calcium is biologically active as ionized calcium, minute changes in the concentration of free, ionized calcium have profound biological effects. Thus, calcium homeostasis is also tightly regulated and because changes in extracellular calcium concentrations can occur very rapidly on the time scale of minutes, regulation has to be almost immediate [17].

### Intestinal and renal control of calcium

Calcium, like Pi, is absorbed mostly by the small intestine through active, channel-mediated pathways and through a passive paracellular route [18–20]. The transcellular pathway is composed of the luminally located TRPV6 Ca^2+^-channel, the intracellular Ca^2+^-buffering protein calbindin D9k, and basolaterally located Na^+^–Ca^2+^-exchanger NCX1 and plasma membrane Ca^2+^-ATPases (PMCA1b) extruding calcium into blood. The paracellular pathway for calcium involves claudins 2, 12, and 15 [18,19,21,22].

In kidney, ionized calcium is almost freely filtered and reabsorbed by different nephron segments [17]. The bulk of calcium is reabsorbed mostly through the paracellular pathway in the proximal tubule driven by solvent drag and a lumen-positive transepithelial potential. The Na^+^/H^+^-exchanger isoform 3 (NHE3) plays an important role in driving solvent drag and its deficiency impairs calcium absorption. The second site of tubular calcium reabsorption is the thick ascending limb of the loop of Henle where calcium together with magnesium is reabsorbed through the paracellular pathway formed by claudins 16 and 19. At last, fine-tuning of calcium reabsorption occurs in the distal convoluted tubule (DCT) and connecting tubule (CNT) where luminally located TRPV5 Ca^2+^-channels mediate the entry step, D28k calbindin buffers intracellular calcium, and NCX1 and possibly PMCA4 export calcium [17]. The activity of these transport proteins in DCT and CNT is tightly regulated by the factors controlling renal calcium reabsorption and are described in more detail below.

Systemic calcium balance is mostly under the control of hormones that also regulate Pi homeostasis. Given the requirement for both calcium and Pi for bone stability and the risk of forming calcium–Pi precipitations in extraosseous sites, this joint regulation is not surprising. However, as dietary supply of both minerals is not always paralleled, the system must also provide for differential regulation of both minerals. The major hormones involved in calcium balance are PTH, calcitriol, and calcitonin [20–22]. Other factors also affect calcium balance such as acid–base status, glucocorticoids, sex hormones, and possibly also FGF23/αKlotho. A fall in ionized serum calcium levels is sensed by the calcium-sensing receptor (CaSR) in parathyroid gland cells and releases the suppression of PTH synthesis and secretion. PTH acts on bone to stimulate osteoclast-mediated calcium release and on kidney to stimulate renal reabsorption of calcium via TRPV5 channels. Last, PTH also stimulates CYP27B1 activity in kidney increasing the 1-α-hydroxylation of calcidiol to the active calcitriol. Calcitriol then synergizes with PTH to stimulate renal calcium reabsorption. In addition, calcitriol also increases intestinal calcium absorption, in part by acting on TRPV6 channels [20–22].

In case of elevated ionized calcium levels, CaSR suppresses PTH secretion and calcitonin is released from C-cells located in thyroid glands. Calcitonin mostly acts on the kidney to increase urinary calcium excretion and on bone to stimulate osteoblast-mediated formation of calcium hydroxyapatite.

### Dysregulated calcium balance in patients with CKD

As discussed later in more detail, CKD is a state of dysregulated mineral homeostasis affecting blood levels of Pi, calcium, and their regulators FGF23/αKlotho, calcitriol, and PTH leading to skeletal abnormalities and extraosseous calcifications including vascular calcifications. These changes are summarized with the term CKD-mineral and bone disorder (CKD-MBD) [17,23–25]. Both, hyper- and hypocalcemia can be present in patients with advanced stages of CKD. Pi retention because of reduced renal clearance can trigger formation of calcium–Pi precipitates lowering ionized calcium levels, which is sensed by the CaSR increasing PTH secretion. PTH then acts on bone to release calcium, which inadvertently increases also Pi release leading to a vicious cycle with continuous bone loss and extraskeletal calcifications with secondary hyperparathyroidism. In addition, it has been shown that FGF23, in an αKlotho-dependent manner, suppresses PTH secretion, which is reduced in CKD and that the CaSR suppressive effect is also reduced because of hyperphosphatemia-related decreased expression of the CaSR. On the other hand, high FGF23 also lowers calcitriol levels, thereby also reducing intestinal calcium reabsorption. However, since renal function is also impaired, net calcium balance remains often even positive. These different interconnected mechanisms have led to an evolution of therapies supplementing calcitriol and calcium, reducing the Pi burden, or to reduce PTH secretion by parathyroidectomy or calcimimetics. These therapies require adaption to the different stages of CKD, the individual setting of patients, and most importantly, often do not fully correct the mineral abnormalities [17,25].

### Mineralization buffers and inhibitors

Under physiological conditions, Pi and calcium in blood are considered near supersaturation [26,27], imposing a risk for spontaneous precipitation and soft tissue calcification. Thus, a mineral buffering system, composed of endogenous local and circulating calcification inhibitors, evolved that prevents extraosseous mineralization and safely allows mineral transport, as reviewed in [28–30]. Briefly, the circulating protein Fetuin-A is able to bind spontaneously occurring calcium–Pi clusters as calciprotein monomers [29,31]. These monomers can form larger aggregates, termed primary calciprotein particles (CPPs), which are rapidly cleared from the circulation by the liver sinusoidal endothelial cells and Kupffer cells [32]. Over time, primary CPPs may undergo transition to secondary CPPs with a crystalline core [28]. The ‘ripening’ of CPPs is utilized in the diagnostic test of serum calcification propensity, measuring the endogenous capacity of serum to prevent the transition of primary to secondary CPPs and, thus, the balance between calcification inhibitors and promoters in the serum [33]. The exact composition and structure of CPPs *in vivo* is still under debate [29,34]. Further mechanisms contribute to an anti-calcific defence against unwanted soft tissue mineralization. A potent calcification inhibitor is inorganic pyrophosphate (PPi), which prevents hydroxyapatite crystal formation and growth [35]. PPi in the vasculature is mainly formed through hydrolysis of ATP by ectonucleotide pyrophosphatase/phosphodiesterase (ENPP) 1 [36] and is degraded to Pi by alkaline phosphatase [37]. Accordingly, both ENPP1 deficiency [38] and vascular overexpression of alkaline phosphatase [39] promotes calcifications in mice. In humans, mutations of ENPP1 can cause idiopathic infantile arterial calcification [40]. Furthermore, vitamin K-dependent proteins like matrix GLA protein [41] and GLA-rich protein [42] serve as inhibitors of extraosseous calcification.

### Pi overload

Phosphate is a natural component in food, occurring both as organic and inorganic phosphate [43]. Pi in meat or dairy products is more bioavailable than organic phosphate in plants, which is found in the form of phytate [44]. Pi is widely used as a food additive in the form of various Pi salts. Pi additives are more effectively absorbed in the intestine, and increase serum Pi more strongly than foods with natural inorganic or organic phosphate content [43]. It has been estimated, that intake of Pi-containing food additives has more than doubled from 1990 to 2012 [45]. Pi additives can increase the food Pi content by up to 60% [46]. Foods without Pi additives have been found to be more expensive than comparable food products with Pi additives [47]. Thus, a too high Pi intake in western diets has been discussed [45]. Currently, the use of Pi additives is very little regulated in the United States or Europe.

In the human general population, higher serum Pi levels are associated with an increased risk of cardiovascular disease (CVD) [48–50]. In men over 60 years, serum Pi has been associated with cardiovascular mortality caused by noncoronary heart disease or stroke [51]. High Pi levels have been also associated with a greater risk of heart failure [52]. Besides cardiovascular mortality, serum Pi levels were recently related to chronic obstructive pulmonary disease mortality [53]. In hospitalized patients, both high and low Pi levels associate with increased in-hospital mortality [54]. After an 11-week-period of high Pi intake in healthy volunteers, blood pressure and pulse rate were increased [55]. Acute Pi load is associated with impairment of endothelial function [56]. Higher serum Pi is in turn associated with microvascular dysfunction [57]. Thus, there is accumulating evidence for a disadvantageous role of Pi in the cardiovascular system.

In mice, detrimental effects of elevated Pi levels were shown in αKlotho-knockout mice, an animal model resembling accelerated aging [58]. However, most insights on Pi as cardiovascular risk factor originate from patients with CKD. CKD is an increasing global health burden, with a prevalence of 9.1% by 2017 [59] and expected to rise in coming years due to the global aging populations and the rise in diabetes and hypertension [60]. In CKD, reduced glomerular function leads to impaired Pi clearance. To minimize Pi retention, FGF23 levels rise early in the course of CKD. This leads initially to a subtle decrease in serum Pi before there is a turnaround and plasma Pi starts to increase at an eGFR of 59.1 ml/min per 1.73 m^2^ [61]. The rate of increase in serum Pi levels before end-stage renal disease (ESRD) is modest, but steady with negligible acceleration [62,63]. The pronounced hyperphosphatemia developing in late-stage CKD and dialysis patients has been recognized as a major determinant of mortality, especially cardiovascular mortality [64].

To explain the association of hyperphosphatemia and cardiovascular mortality, Pi has been increasingly associated with pro-inflammatory effects [65–67]. Increased vascular inflammation in the absence of atherosclerotic disease is observed in patients with CKD [68]. An association of Pi levels and inflammatory markers such as C-reactive protein (CRP) and IL-6 was noted in patients with CKD [69]. High Pi diet (1.2%) in rats with adenine-induced CKD elevates TNF levels and oxidative stress markers [70]. In dialysis patients, higher CRP levels are predictive for cardiovascular events [71], while the use of Pi binders is associated with reduced levels of the inflammatory markers CRP and IL-6 [72]. However, Pi binders failed to lower serum Pi levels and attenuate aortic stiffness in CKD stage 3b–4 patients [73]. Nevertheless, Fetuin-A could be down-regulated during inflammation and was negatively associated with CRP levels and increased all-cause and cardiovascular mortality in hemodialysis patients [74]. Thus, low Fetuin-A levels might contribute to ectopic calcification in these patients [74].

## Inflammation and vascular calcification in hyperphosphatemia

### Vascular calcification—a critical sequelae of Pi overload

The mechanisms, how Pi is linked to cardiovascular events and mortality is not completely understood. A putative link between disturbed Pi metabolism and cardiovascular events is the development of vascular calcification [65]. Numerous studies associate the presence of vascular calcification in large arteries with increased cardiovascular risk in CKD [75], diabetes mellitus [76], or the aging general population [77]. Vascular calcification can be localized in the medial or intimal layers of the arteries. Intimal calcification is associated with atherosclerotic processes, while medial calcification is observed also in the absence of lipid deposits and occurs diffusely in the arterial medial layer [78]. Intimal and medial calcification exhibit different risk factor profiles [79]. Pi has been especially linked to medial vascular calcification [80]. How and if medial calcification is causally linked to cardiovascular mortality remains to be fully established [81]. A critical consequence of medial vascular calcification could be arterial stiffening, leading to increased pulse pressure and left ventricular hypertrophy (LVH) [82]. In turn, higher Pi levels are associated with a higher ankle-brachial index as indicator of arterial stiffness [83].

### Mechanisms of medial vascular calcification

**Figure 2:**
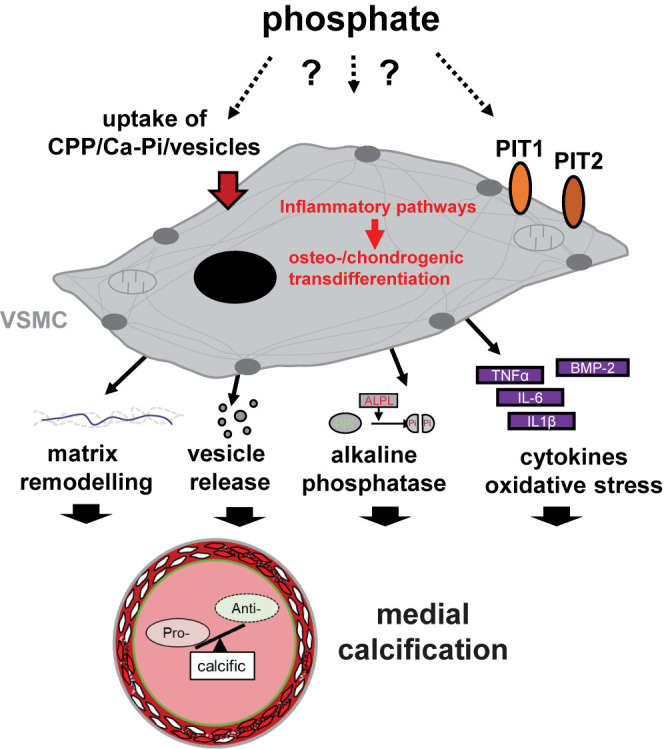
Pro-calcific effects induced by Pi on VSMCs The exact mechanisms underlying the effects of Pi on VSMCs are not yet established, but may involve Pi transport by PiT1 and PiT2, as well as cellular uptake of CPPs, calcium-Pi nanocrystals or pro-calcific vesicles. Exposure to elevated Pi conditions activates pro-inflammatory pathways in VSMCs to promote an osteo-/chondrogenic transdifferentiation. These cells induce various pro-calcific effects, involving remodeling of the extracellular matrix, release of vesicles and apoptotic bodies, inactivation of calcification inhibitors such as pyrophosphate (PPi) by tissue-nonspecific alkaline phosphatase (ALPL), and release of various pro-inflammatory and pro-calcific transmitters and oxidative stress. Thus, a pro-calcific environment in vascular tissue may ensue, augmenting medial vascular calcification.

The development of medial vascular calcification is a complex and regulated process, culminating in the deposition of calcium–Pi complexes in arteries. A critical role in this process is attributed to vascular smooth muscle cells (VSMCs; Figure 2) [66]. Although the underlying mechanisms may show differences, VSMCs are also considered critical contributors to intimal calcification [84]. VSMCs originate from diverse embryonic tissues and retain functional plasticity [85]. Upon exposure to calcification stimuli, most notably Pi, VSMCs transdifferentiate into cells with some osteoblastic and chondroblastic characteristics [66]. The pro-calcific transdifferentiation is characterized by increased expression of osteogenic and chondrogenic transcription factors and enzymes [66,86]. Epigenetic effects are involved in VSMCs transdifferentiation [87]. A key role is attributed to the osteogenic transcription factor core-binding factor α1 (CBFA1, also known as RUNX2), which is up-regulated in VSMCs upon Pi exposure [65,88]. Deficiency of CBFA1 in VSMCs inhibits vascular calcification in mice [89]. The phenotypical change of VSMCs precedes the onset of calcification [90]. Osteo-/chondrogenic transdifferentiated VSMCs augment calcification by various mechanisms. The production of tissue-nonspecific alkaline phosphatase, which cleaves and inactivates the extracellular calcification inhibitor PPi, is considered a decisive mechanism [36]. Furthermore, osteo-/chondroblast-like VSMCs release calcifying vesicles [91], elastin-degrading proteases and increase collagen I and fibronectin deposition in the extracellular matrix [66,92]. In turn, VSMCs are able to sense matrix stiffness, which stimulates the calcification process [93]. Calcifying VSMCs produce various osteogenic and pro-inflammatory cytokines [66]. Besides VSMCs, other cells modulate vascular calcification [94] and various cell types are able to calcify *in vitro* [95]. In valvular calcification, valvular interstitial cells promote calcification [96], a process that is augmented by Pi [97]. Thus, upon Pi exposure, a pro-calcific microenvironment develops, fostered by cell-mediated processes [66].

Exposure of VSMCs to elevated Pi concentrations is sufficient to cause vascular calcification *in vitro* [86]. The detrimental effects of Pi may require formation of calcium-Pi nanocrystals [98]. Both, calcium or Pi exposure are able to induce VSMC calcification and act synergistically [99,100]. Thus, calcium has also been considered a critical factor in vascular calcification [99]. Complex signalling pathways are activated in VSMCs upon exposure to elevated Pi levels leading to osteo-/chondrogenic transdifferentiation and subsequent calcification [66]. A key role is played by the type III sodium-dependent Pi transporters (PiT1/SLC20A1 and PiT2/SLC20A2) [101–103]. While PiT1 augments vascular calcification through Pi transport-dependent and -independent roles [101,102], PiT2 retains a protective role [103] during phosphate exposure. Uptake and lysosomal processing of calcium-Pi crystals in VSMCs was discussed [104]. Vesicles from calcifying VSMCs augment recipient VSMC calcification [105]. Moreover, secondary CPPs may be taken up by VSMCs to directly trigger intracellular signalling and promote calcification [106]. However, the exact mechanism, how Pi modulates VSMC functions remains to be established.

### Inflammatory pathways in Pi-induced vascular calcification

Vascular calcification is paralleled by local up-regulation of pro-inflammatory responses in the arterial wall [107] and inflammatory cytokines augment the development of vascular calcification [70]. A local microinflammation alongside medial calcification was described in CKD patients [107]. Furthermore, systemic inflammatory responses may modulate effects of Pi on VSMCs [70]. Pi overload may directly induce vascular inflammation *in vitro* and *in vivo* [70]. The signalling controlling osteo-/chondrogenic transdifferentiation and calcification of VSMCs promoted by Pi involves activation of pathways associated with inflammation in VSMCs [66].

Pi exposure activates the pro-inflammatory transcription factor nuclear factor κ-light-chain-enhancer of activated B cells (NF-kB) in VSMCs, a critical step augmenting osteo-/chondrogenic transdifferentiation [108]. Pi-induced activation of NF-kB involves regulation of the inhibitor of nuclear factor κ B (IkB) kinase (IKK) complex by the serum- and glucocorticoid-inducible kinase SGK1 [109]. Interestingly, IKKβ is involved in an anti-calcific effect, which is independent of kinase function and involves β-catenin [110]. NF-kB induces tristetraprolin expression, which destabilizes the transmembrane protein ankylosis protein homolog (*ANKH*) mRNA, thus, reducing extracellular PPi levels [111]. In addition, upon Toll-like receptor 2 (TLR2) activation, NF-kB represses expression of osteoprotegerin [112], which inherits a complex role in vascular diseases and may inhibit vascular calcification [113]. In accordance with these findings, a pro-calcific function during Pi exposure was also shown for transforming growth factor β-activated kinase 1 (TAK1), an upstream regulator of NF-kB [114]. Also, the receptor activator of NF-kB ligand (RANKL)/RANK system augments vascular calcification through NF-kB [115]. Further NF-kB activators, such as the pro-inflammatory cytokines TNF [111,116] or TNF-related weak inducer of apoptosis (TWEAK) [117] augment vascular calcification. Pro-calcifying conditions induce expression of TNF in VSMCs [106] and TNF stimulates alkaline phosphatase expression via NF-kB-mediated MSX2 expression [118]. TNF may further promote vascular calcification via endoplasmic reticulum stress through activation of C/EBP homologous protein [119]. Interestingly, the pro-calcific effects of TNF on alkaline phosphatase may be dependent on the cell type [120]. Contrary to the pro-calcific effects in VSMCs, inhibition of NF-kB in osteoblasts maintains bone formation [121]. Hypothetically, chronic inflammatory processes could link bone demineralization and vascular calcification via NF-kB [122]. Inhibition of NF-kB ameliorates ovariectomy-induced bone loss in mice [123] and Pi-induced vascular calcification in a CKD mouse model [124]. Additional various activators of NF-kB augment vascular calcification, such as TLR4 activation [125], palmitic acid [126], indoxyl sulfate [127], IL-18 [128], atmospheric ultrafine particles [129], or trimethylamine N-oxide [130].

*In vitro* exposure to high Pi conditions enhances release of the pro-inflammatory cytokine IL-6 in VSMCs [125]. IL-6 expression in VSMCs is promoted also by TNF [131], an effect involving activator protein 1 (AP-1) [132]. IL-6 promotes CBFA1 expression in VSMCs through signal transducer and activator of transcription 3 (STAT3) [133]. IL-6 is involved in Pi-induced VSMC senescence via p53/p21 [134]. Senescence and the senescence-associated secretory phenotype were recently discovered as a decisive mechanism contributing to vascular calcification [135–137]. Moreover, senescence is linked to oxidative stress [135]. Pi exposure itself is able to generate oxidative stress in VSMCs, and inhibition of oxidative stress blunts VSMC-calcification [108,138,139]. Oxidative stress promotes DNA damage inducing a DNA damage response [137]. Components of the DNA damage response, such as ataxia telangiectasia-mutated (ATM) protein or poly adenosine diphosphate (ADP) ribose polymerase were recently linked to vascular calcification [137,140,141]. Presumably as a protective mechanism, the transcription factor nuclear factor erythroid 2-related factor 2 (NRF2) induces expression of antioxidant and anti-inflammatory genes in response to Pi-induced oxidative stress [142]. Accordingly, activation of NRF2 has been associated with anti-calcific effects [142,143]. Senolytic compounds and NRF2 agonists were therefore discussed as therapeutic concepts in vascular calcification [144].

A variety of other signalling molecules were associated with vascular calcification, most notably the bone morphogenic proteins from the transforming growth factor β (TGFβ) signalling family [145]. Also, CRP is increased in calcifying vascular tissues and promotes Pi-induced vascular calcification [146]. In addition, upon Pi exposure, an up-regulation of the NALP3 inflammasome complex and IL-1β release was observed [147]. IL-1β promotes senescence and osteogenic transdifferentiation of VSMCs [148]. Furthermore, cytosolic [149] and lipoprotein-associated [150] phospholipase A2 may contribute to cardiovascular calcification. Epoxyeicosatrienoic acids were shown to augment [151], while prostaglandin E2 inhibits vascular calcification [152]. Further pro-inflammatory mediators associated with vascular calcification include IL-8 [153], IL-24 [154] or IL-18 [128]. Also, angiotensin II and aldosterone were associated with pro-calcific effects [155]. Some inflammatory pathways may also be context-dependent. The adipokine chemerin was associated with pro-inflammatory effects in endothelial cells [156], but inhibits vascular calcification [157].

Another link between Pi metabolism, kidney function, and vascular inflammation may be αKlotho. αKlotho deficiency in mice results in widespread ectopic calcification, mostly attributed to excessive plasma Pi [158]. Also, in zebrafish, αKlotho deficiency promotes osteogenic transdifferentiation and ectopic calcification [159]. Besides its role as renal co-receptor for FGF23, αKlotho is also cleaved from the membrane and found in the systemic circulation [160]. Soluble αKlotho inhibits VSMC Pi uptake and prevents vascular calcification *in vitro* [160]. Reduction of soluble αKlotho is associated with higher levels of pro-inflammatory cytokines in patients with CVD [161]. Soluble αKlotho interferes with the pro-fibrotic effects of TGFβ1, angiotensin II and Pi [162]. Mechanistic effects of αKlotho include inhibition of PI3K signalling [163], NF-kB [164], transient-receptor potential canonical Ca^2+^ channel 1 [165] and activation of NRF2 [166]. However, soluble αKlotho may also directly impact on Pi homeostasis [167], and a vascular expression of αKlotho remains controversial [168,169]. Similarly, the direct effects of FGF23 on vascular calcification are ill defined [170,171].

## Role of FGF23 in Pi overload/toxicity

### FGF23 in CKD and the general population and its link to CVD and inflammation

The early rise of FGF23 in the course of CKD is important to minimize Pi retention while kidney function declines. In CKD patients of the Chronic Renal Insufficiency Cohort (CRIC), cFGF23 rise when eGFR fall below 60 ml/min 1.73 m^2^ [61] with a significant increase in the slope 5.1 years before reaching ESRD. Similar, plasma Pi and PTH, which both increase continuously with a significant change in slope 5.2 and 4.9 years before patients were reaching ESRD, respectively [62]. Interestingly, cFGF23 has a much steeper slope compared with Pi and PTH and is the only parameter with a pronounced increase in acceleration before reaching ESRD [62]. The accelerated rise in cFGF23 goes along with higher use of vitamin D analogues and Pi binders [62]. In different ESRD cohorts, it has been shown that patients with higher plasma FGF23 levels have an increased odds ratio or hazard risk for death independent of plasma Pi levels [172,173]. Elevated cFGF23 levels are not only found in patients with ESRD, but also with CKD stages 2–4 as well as in the general population and are an independent risk factor for mortality [174–176]. The association between cFGF23 and mortality was not confounded or modified by plasma Pi [174]. And although PTH and FGF23 correlated similar with eGFR, their correlation with mortality was different [174]. Interestingly, the use of FGF23 as biomarker in combination with the N-terminal pro-brain natriuretic peptide (NT-pro-BNP), as a marker of cardiac stretch and myocyte injury, high sensitivity CRP (hs-CRP), as a marker for chronic inflammation and cystatin C, as a marker for kidney function, significantly improved the prediction of death in a cohort of patients with advanced CKD [177]. In the CRIC cohort, plasma cFGF23 improved the risk prediction for all-cause mortality and heart failure hospitalizations for patients with mild to moderate CKD, but not cardiovascular mortality, ESRD or atherosclerotic events [178].

**Figure 3:**
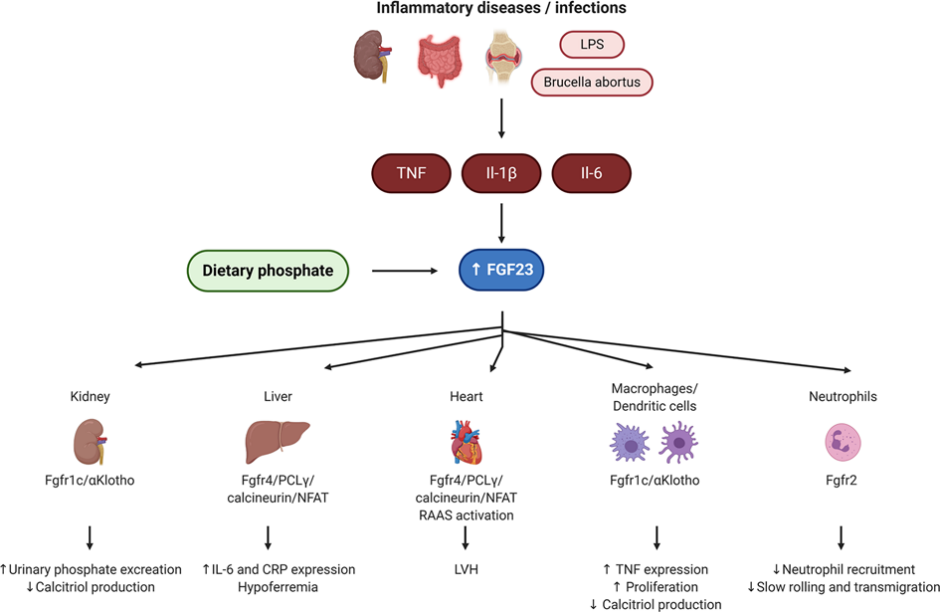
Summary of the regulation of FGF23 by inflammatory stimuli and dietary Pi and actions of FGF23 in the context of inflammation (Created with BioRender.com) Inflammation in various organs such as kidney, intestine or bone as well as systemic inflammation induced by LPS or bacteria such *Brucella abortus* increase pro-inflammatory cytokines (i.e. TNF, IL-1β, IL-6), which stimulate FGF23. Likewise, dietary Pi can also stimulate FGF23. FGF23 acts on kidney to reduce Pi reabsorption and calcitriol levels, on liver to cause inflammation, on heart potentially to induce LVH, and on immune cells to affect cytokine production, proliferation, and recruitment. Abbreviation: LPS, lipopolysaccharide.

Thus, mechanisms beyond mineral metabolism and renal function may be responsible for the association between elevated cFGF23 levels and mortality. The possible involvement of FGF23 in CVD and inflammation is often used to explain the link between FGF23 and mortality (Figure 3). In CKD patients as well as in two elderly population cohorts, higher cFGF23 levels are independently associated with increased left ventricular mass index and LVH [172,179,180]. Interestingly, children with heart failure have elevated plasma cFGF23 compared with healthy controls, which correlated independent of eGFR with NT-pro-BNP and end left ventricular end-diastolic diameter [181]. In patients with advanced CKD or incident dialysis, high cFGF23 levels are associated with an increased hazard ratio for cardiovascular events independent of established risk factors for CVD and abnormalities of mineral metabolism [176,182]. In incident dialysis patients, there was no differences in pulse-wave velocity and abdominal aortic calcification dependent on cFGF23 [182]. In the community based Northern Manhattan Study, each unit increase in log cFGF23 was associated with a greater odds of total carotid plaque area after adjusting for eGFR, sociodemographic, vascular risk factors and mineral metabolites [183]. Furthermore, in CKD and healthy control subjects, the presence of aortic calcification, the abdominal aortic calcification score as well as an abnormal pulse wave velocity (≥10 m/s) were independently associated with higher iFGF23 [184]. Whereas in hemodialysis patients, high plasma iFGF23 levels were associated with higher coronary artery calcification score independent of serum Pi [185]. In the multicentre prospective osteoporotic fractures in Men cohort (MrOS), iFGF23 was not associated with all-cause mortality and CVD-related deaths [186]. Only in a sub-population with eGFR > 60 ml/min/1.73 m^2^, the hazard ratio for cardiovascular mortality was increased for higher iFGF23 [186]. However, the overall mortality rate due to CVD was low (37%) in the MrOS cohort and included heart failure, stroke, myocardial infarction, pulmonary embolism, and arrhythmias for which the association with iFGF23 may be weaker or completely absent [186]. Also, in a small Japanese cohort of hemodialysis patients, cFGF23 is increased, but is neither associated with cardiac dysfunction nor atherosclerosis [187]. In hemodialysis patient with secondary hyperparathyroidism, higher baseline serum FGF23 levels are associated with an increased risk for the primary outcome (death, first myocardial infarction, hospitalization for unstable angina, heart failure or peripheral vascular event) [188]. The administration of the calcimimetic cinacalcet to those patients lowers serum FGF23 within 20 weeks, which was maintained throughout the study (100 weeks). Lower FGF23 levels in the treatment arm are associated with lower risk of cardiovascular mortality and cardiovascular events such as heart failure and sudden cardiac deaths [188]. Thus, conflicting data exist whether FGF23 associates with cardiovascular outcomes and these differences may depend on different cohorts studied, different end points, or on other unadjusted variables that modify a link between FGF23 and specific cardiovascular end points.

Elevated plasma FGF23 levels have been linked to inflammatory cytokines and active disease states of autoinflammatory diseases in patients with CKD or other inflammatory diseases. In the CRIC cohort, higher cFGF23 levels are associated with higher levels of the inflammatory markers CRP, IL-6, TNF, and fibrinogen and display higher odds ratio for severe inflammation independent of mineral metabolism and renal function [189]. Strength of association between IL-6 and CRP with death prior ESRD was only modestly attenuated by adjustment for cFGF23. Hence, FGF23 and inflammation may have most likely distinctive downstream effects that account for their additive impact on mortality [190]. Additionally in the CRIC cohort, higher cFGF23 levels are associated with increased hazard ratios for infections as well as for the risk of hospitalization with major infections [191]. In hemodialysis patients, serum iFGF23 is not correlated with hs-CRP or IL-6, but is an independent predictor of the risk of infection [192]. The association between FGF23 and inflammation markers is not limited to CKD. In the Swiss Kidney Project on Genes in Hypertension (SKIPOGH), iFGF23 positively correlates with TNF in the general population [193], whereas the Reasons for Geographic and Racial Differences in Stroke study found a positive correlation of cFGF23 with IL-6 and IL-10 in a non-CKD population [194]. In an elderly population, i- and cFGF23 are associated with inflammation as determined by hs-CRP [195]. Furthermore, several inflammatory diseases such as inflammatory bowel disease (IBD), osteoarthritis, axial spondylorarthritis, lupus nephritis, psorasis, and AKI are associated with elevated FGF23 levels [196–201]. For example, chondrocytes from patients suffering from osteoarthritis have elevated *FGF23* gene expression levels [197], while children, who were in an acute phase of IBD, have elevated circulating FGF23 levels that decreased again in the remission phase [196]. Interestingly, these children also have an increase in calcitriol levels in the acute IBD phase compared with the remission phase and to control subjects. In active lupus nephritis, serum iFGF23 is associated with IL-6, TNF and urinary monocyte chemoattractant protein 1 (MCP-1) of which only MCP-1 remains significant after adjustment for calcidiol. No data are available about the previous use of corticosteroids in this cohort [199]. The regulatory mechanism of FGF23 expression in inflammatory disease as well as its physiological function in the regulation of inflammation and the impact on mortality in this context is barely understood.

Taken together, in various cohorts, associations between FGF23 and CVD or inflammation were found, but if lowering FGF23 in CKD could reduce the FGF23-associated all-cause, cardiovascular mortality or risk for infection is unknown. Directly targeting FGF23 in CKD is unfavorable as blocking iFGF23 with neutralizing antibodies in a 5/6-nephrectomized CKD rat model reduced survival due to a severe increase in Pi levels and concomitant vascular calcification, although secondary hyperparathyroidism was decreased, calcitriol levels increased, and multiple bone parameters improved [14]. Furthermore, these CKD rats develop left ventricular changes independent of the FGF23 antibody treatment [14]. Similar in osteoblast/osteocyte-specific *Fgf23*-knockout mice (*Fgf23^fl/fl^ Dmp-cre*), induction of CKD by adenine diet resulted in higher serum Pi and blood urea nitrogen as well as increased aortic calcification and more pronounced cardiac hypertrophy compared with wild-type mice with adenine-induced CKD [202]. It has been shown that Pi by itself is an independent risk factor for mortality in ESRD patients [203]. These animal studies underline the importance of FGF23 in the regulation of plasma Pi levels in advanced CKD and the concomitant maintenance of normal cardiac homeostasis. Hence, it is important to investigate not only associations, but also biological mechanism to clarify the role of FGF23 in both CVD and inflammation and thereby find possible biological targets to reduce the FGF23-associated mortality. Moreover, some of these studies clearly point to FGF23-independent mechanisms causing vascular calcification and LVH and associate these changes with Pi or inflammatory factors.

### FGF23 and cardiac hypertrophy

The mechanisms behind the association of FGF23 and cardiovascular risk have been investigated *in vitro* and *in vivo*. It has been shown that FGF23 increases cell surface area of mouse HL-1 cardiomyocytes in a dose-dependent manner after 48 h [204]. In mouse ventricular muscle strips *Fgfr* 1–4 as well as *αKlotho* mRNA expression is detected [204]. FGF23 treatment of ventricular muscle strips activates mitogen-activated protein kinase (MAPK) signaling and increases *Egr1* mRNA expression [204]. At the same time, markers of LVH, atrial natriuretic peptide (*Anp*), brain natriuretic peptide (*Bnp*) and β-myosin heavy chain (*Myh7*) mRNA expression are upregulated in response to FGF23 [204]. Furthermore, FGF23 transiently increases intracellular calcium levels of primary cardiomyocytes and contractile force in *ex vivo* ventricular muscle strips [204]. In neonatal rat ventricular cardiomyocytes (NRVCs), FGF23 increases cell surface area and α-actinin, indicative for increased sarcomeric content, in a dose-dependent manner similar to the well-known hypertrophic factor FGF-2 [205]. Furthermore, the cardiac hypertrophic markers *Mhy7, Anp*, and *Bnp* are increased, while α myosin heavy chain (*Mhy6*) and medium chain acyl-CoA dehydrogenase (*Mcad*) are decreased in NRVCs upon FGF23 treatment [205]. The effect of FGF23 on NRVCs is FGFR-dependent, but αKlotho-independent and involves PLCy and calcineurin-NFAT signalling [205]. Systemic and myocardial administration of FGF23 in 12-week old C57BL/6 mice induces cardiac hypertrophy [205]. In both models, FGF23 increases heart weight to tibia length, left ventricular free wall thickness, cross-sectional surface area of individual cardiomyocytes and *Mhy7, Anp*, and *Bnp* mRNA expression, while it decreases *Mhy6* and *Mcad* mRNA expression [205]. Additionally, in mice treated with myocardial FGF23 injections, there is no change in cardiac ejection fraction, but left ventricular internal diameter in diastole is decreased while relative wall thickness (ratio of wall thickness to internal chamber diameter) is increased [205]. In contrast, two other studies have shown no effect of FGF23 excess on heart weight per tibia length or body weight. In one study, systemic administration of FGF23 to 13-week-old C57BL/6 mice had no effect on heart weight per tibia length [206]. In another study, myocardial injection of an adeno-associated virus carrying the FGF23 sequence led to cardiac overexpression of FGF23, but did not induce cardiac hypertrophy [207]. The discrepancy between these studies may be due to the different routes of administration or different dosages.

By utilizing cardiomyocytes of *Fgfr4*-knockout mice and specific Fgfr4-neutralizing antibodies to block FGF23 signaling, Fgfr4 has been revealed as a crucial receptor for FGF23 signalling in the heart [11]. Cardiac Fgfr4 activation by FGF23 is independent of αKlotho and signalled via phospholipase C γ (PLCγ) /calcineurin/NFAT signaling pathway to induce cardiomyocyte hypertrophy [11]. The development of LVH is prevented in *Fgfr4*-knockout mice fed a high Pi diet as well as in 5/6-nephrectomized rats treated with anti-Fgfr4 antibodies [11]. Furthermore, administration of recombinant FGF23 to wild-type mice leads to LVH, which is prevented in cardiomyocyte-specific *Fgfr4*-knockout mice as shown by reduced heart weight per body weight ratio and down-regulation of hypertrophic marker genes *Anp* and *Bnp* [208]. Moreover, co-administration of FGF23 and soluble αKlotho prevents LVH by activating MAPK instead of PLCy signalling pathway [208]. *Fgfr4*-knockin mice have elevated FGF23 levels and spontaneously developed LVH, but the degree of LVH is not correlated to plasma FGF23 levels [11]. Hence, Fgfr4 activation alone independent of FGF23 is able to induce LVH. Nevertheless, in a small study cohort with deceased CKD patients who received renal replacement therapy before the age of 15 years, 67% of patients had LVH at the time of death. The patients with LVH were significantly older and spent longer time on renal replacement therapy [209]. Cardiac FGF23 and FGFR4 expression are higher in patients with LVH compared with patients without LVH [209]. Furthermore calcineurin-NFAT signalling pathway is activated as shown by increased mRNA expression of calcineurin subunit B expression as well as nuclear localization of the activated NFAT in CKD patients with LVH compared with patients without LVH [209].

As discussed above, several CKD animal models have been analyzed to investigate the role of FGF23 in LVH. In adenine diet-induced CKD rats, elevated plasma FGF23 goes along with vascular calcification and increased pulse pressure, pulse-wave velocity as well as increased left ventricular mass per body weight ratio [210]. Both, FGF23 and pulse pressure are associated with left ventricular mass per body weight ratio [210]. In 5/6-nephrectomized CKD rats fed a high Pi diet, LVH is prevented by the administration of the Pi binder lanthanum. Lanthanum normalizes serum Pi levels and reduces serum iFGF23 by half, sufficient to prevent LVH [211]. Not only lowering Pi levels, but also increasing calcitriol levels impacts on the development of LVH in 5/6-nephrectomized CKD rats. Six weeks of calcitriol (300 ng/kg BW/day) treatment of 5/6-nephrectomized rats with LVH increased *Fgf23* mRNA expression in bone, while in the heart FGF23 protein and *Fgfr4* mRNA expression, cardiomyocyte cross-sectional area as well as the expression of the hypertrophic markers *Mhy7* and *Bnp* is reduced [212]. Furthermore, calcineurin protein levels are reduced by calcitriol treatment, while NFAT phosphorylation is increased preventing NFAT translocation to the nucleus [212]. Co-treatment of neonatal rat ventricular myocytes (NRVMs) with recombinant FGF23 and calcitriol prevents the increase in cross-sectional area of NRVMs as well as the interaction of Fgfr4 with PLCγ [212]. This suggests that calcitriol counteracts the pro-hypertrophic effect of FGF23 by interfering with the FGF23/Fgfr4/calcineurin/NFAT signalling pathway [212].

*Col4a3*-knockout mice, a CKD mouse model with FGF23 excess, have increased *Anp* and *Myh7* mRNA expression and experiences a decrease in left ventricular function as determined by decreased fractional shortening and ejection fraction [204]. The cardiac hypertrophy phenotype of *Col4a3*-knockout mice is dependent on the genetic background. *129SV-Col4a3*-knockout mice do not develop cardiac hypertrophy *per se*, but show an increase in hypertrophic gene markers and reduced cardiomyocyte contractility despite the absence of cardiac hypertrophy [204,213]. In contrast, *B6-Col4a3*-knockout mice develop cardiac hypertrophy with simultaneous up-regulation of the hypertrophic markers, *Anp, Bnp*, and *βMhc* [213]. The reason for the background-specific differences may lay in the temporal differences in progression of CKD in the two mouse strains. Interestingly, overexpression of dentin matrix protein (*Dmp1*) in *B6-Col4a3*-knockout mice lowers plasma c- and iFGF23 levels and prevents LVH determined by cardiomyocyte area and perimeter, left ventricular mass and left ventricular posterior wall thickness despite no change in hypertension, blood pressure and kidney function and in the presence of persistent hyperphosphatemia [214].

In contrast with the rodent models discussed above, in *Fgf23*-transgenic (*Tg*) mice, a model of FGF23 excess, secondary hyperparathyroidism, hypophosphatemia and hypotension, high FGF23 is accompanied by increased left ventricular mass, interventricular septum thickness and left ventricular posterior wall thickness [215]. In these mice, LVH is prevented by a high Pi diet [215].

Models of FGF23 excess such as *αKlotho*-knockout mice, *Col4a3*-knockout mice, *Fgf23-Tg* mice, 5/6-nephrectomized rats and spontaneously hypertensive rats (SHRs) develop cardiac hypertrophy, but these models develop simultaneously hypertension, hyperphosphatemia or secondary hyperparathyroidism, which by themselves may lead to LVH [204,205,213,216]. Interestingly, non-CKD and non-hypertensive models with FGF23 excess such as *Hyp* or *Phex-C733R* mice, both models with mutation in the *Phex* gene and consequently excessively high FGF23 levels, do not display cardiac hypertrophy [217–219]. Patients with FGF23-related hypophosphatemic disease such as tumor-induced osteomalacia (TIO) and X-linked hypophosphatemic rickets (XLH) do not develop LVH nor do iFGF23 significantly correlated with parameters of LVH [220]. In pediatric patients with XLH, but normal renal function, plasma iFGF23 does not correlate with left ventricular mass index [221]. In experimental heart hypertrophy induced by transverse aortic constriction in wild-type and *Fgf23/Vdr*-double knockout mice, hypertrophic remodelling, left ventricular functional impairment or left ventricular fibrosis developed independent of the presence of FGF23 [222]. Hence, FGF23 alone may not be sufficient to cause cardiac hypertrophy, but a combination of factors and perhaps a specific microenvironment induced by CKD is needed.

More recently another possible pathway of how FGF23 is contributing to the development of LVH involving angiotensin II was described. Recombinant FGF23 increases renin–angiotensin–aldosterone system (RAAS)-associated gene expression and angiotensin II abundance in NRVMs and neonatal rat cardiac fibroblasts (NRCFs) [223,224] and thereby indirectly leads to increased cardiomyocyte hypertrophy via angiotensin II-dependent inositol trisphosphate-mediated Ca^2+^ signalling pathway [224]. Similar to the FGF23-mediated cardiac hypertrophy via induction of calcineurin/NFAT pathway, the activation of RAAS by FGF23 in NRVM causes cardiomyocyte hypertrophy, which can be blocked by the angiotensin receptor blocker losartan, and the mineralocorticoid receptor antagonist spironolactone [223].

## FGF23 and inflammation

### Regulation of FGF23 by inflammatory cytokines

Recently, there is growing evidence that FGF23 expression is regulated by inflammatory stimuli and cytokines such as lipopolysaccharide (LPS), TNF, IL-1β, and IL-6 [193,225–229]. Administration of low dose and chronic intermittent dose of LPS in wild-type mice transiently increases plasma FGF23 with marked induction of *Fgf23* mRNA expression in spleen [228,230]. In both, activated peritoneal macrophages and bone marrow-derived dendritic cells (BMDCs), *Fgf23* mRNA expression is up-regulated by LPS. Blocking NF-kB prevents LPS-dependent up-regulation of *Fgf23* mRNA [228]. The deletion of a −16 kb enhancer element of the *Fgf23* gene attenuates the increase in *Fgf23* mRNA expression in bone, bone marrow, spleen, thymus, kidney, intestine, lung and liver as well as the rise in plasma iFGF23 in response to a high dose of LPS-, or the inflammatory cytokines IL-1β and TNF [231]. Furthermore, the −16 kb enhancer element of *Fgf23* gene contributes to the rise in iFGF23 in the oxalate nephropathy mouse model and deletion of the element leads to a decrease in plasma iFGF23 levels [231].

A single injection of heat inactivated *Brucella abortus* (BA) or IL-1β in wild-type mice increases plasma cFGF23, but not iFGF23 levels paralleled by increased *Fgf23* mRNA expression in bone [225]. In the *Col4a3*-knockout mice with mild CKD, a single injection of IL-1β increases cFGF23 similar to wild-type mice, while iFGF23 is increased approximately ten-times more in response to IL-1β compared with wild-type mice [225]. However, inhibition of IL-1β by Rilonacept in CKD stage 3–4 patients had no direct effect on FGF23 nor other parameters of mineral metabolism [232]. In a congenital mouse model of CKD (*Ebf1^fl/fl^, Foxd1-cre* mice), IL-1β is the only cytokine with increased production in the kidney and elevated levels in the circulation [233]. Treatment of *ex vivo* bone chips cultured with anti-IL-1β antibodies in the presence of *Ebf1^fl/fl^, Foxd1-cre+* mouse serum prevents the upregulation of FGF23 [233]. Similarly, treatment of *Ebf1^fl/fl^, Foxd1-cre+* mice with anti-IL-1β neutralizing antibodies attenuates the rise in plasma FGF23 [233].

The inflammatory cytokine TNF stimulates *Fgf23* mRNA expression in primary osteocytes and elevated plasma iFGF23 levels in wild-type mice [193]. In CKD animal models, renal inflammation is evident by increased expression of inflammatory cytokines and increased phosphorylation of NF-kB [193]. Administration of anti-TNF-neutralizing antibodies in CKD mice (*Pkd1* conditional-knockout mice and oxalate nephropathy) as well as in the *Il10*-knockout mice a non-renal inflammation model of colitis normalizes elevated plasma iFGF23 levels [193]. In wild-type mice fed a high-fat diet for 3 weeks, plasma iFGF23 levels are increased, which is blunted in *Tnf*-knockout mice [234]. In children with Crohn’s disease, anti-TNF therapy lowers inflammatory cytokines TNF and IL-6 and increases plasma calcitriol and PTH, but not iFGF23 levels [235]. However, disease activity upon treatment was mild and no data about plasma Pi levels were available. Based on higher calcitriol levels, PTH might increase in response to elevated Pi levels, while the rise in iFGF23 is prevented by anti-TNF treatment.

Plasma FGF23 rises rapidly after onset of folic acid-induced AKI (FA-AKI) in wild-type mice and is accompanied by increased *Fgf23* mRNA expression not only in bone, but also in spleen, thymus, and heart [13,236]. In parallel to plasma FGF23, there is a rise in plasma cytokines such as IL-5, IL-6, IL-10, IFNγ, TNF, and CXC chemokine ligand 1 (CXCL1)/keratinocyte chemoattractant (KC) [226]. Anti-inflammatory treatment with dexamethasone in mice with FA-AKI and ablation of IL-6 in mice with adenine-high Pi diet-induced CKD attenuates the rise in plasma iFGF23 [226].

In 5/6-nephrectomized rats, the magnitude of rise in plasma FGF23 largely depends on dietary Pi content [237]. However, adding an additional inflammatory stimulus like LPS further increases plasma FGF23 levels in 5/6-nephrectomized rats on low Pi diet [237].

Taken together several inflammatory cytokines stimulate FGF23 expression, which can be blocked by neutralizing antibodies or anti-inflammatory treatment. Further studies have to elucidate if these cytokines are orchestrated or act independently on the elevation of FGF23 expression.

### FGF23 as regulator of inflammation

The function of FGF23 in the immune system is unknown. However, microarray analysis of three different mouse models of FGF23 excess (*Col4a3*-knockout, *Hyp*, and *Fgf23-Tg* mice) revealed activation of genes regulating inflammation such as *Tgfb1, Tnf, Il-1b*, and *Nf-kb* [238].

The human hepatocellular carcinoma cell line HepG2 and primary mouse hepatocytes express high levels of FGFR4, but no αKlotho [239]. HepG2 cells activate upon treatment with cell supernatants of FGF23-overexpressing HEK293 cells the Fgfr4/PLCγ/calcineurin/NFAT signalling pathway [239]. Furthermore, FGF23 increases CRP and IL-6 expression, which is blocked by anti-Fgfr4 or cyclosporin A treatment [239]. Twice daily injections of FGF23 increases hepatic *Crp* and *Il6* mRNA expression and CRP protein levels in wild-type mice [239]. Similar results have been shown in mouse models of FGF23 excess (αKlotho-hypomorphic mice, high Pi diet-induced elevation of FGF23 and 5/6-nephrectomized mice) [239]. However, the increase in CRP and IL-6 in these models, although Fgfr4 and calcineurin-dependent, is not necessarily due to FGF23 signalling.

FGF23 has αKlotho-independent effects in murine neutrophils, which mainly express Fgfr2, but not αKlotho [240]. Intratracheal injection of viable *Escherichia coli* bacteria leads to pneumonia in mice, which activates the immune system to recruit neutrophils to lungs and spleen to prevent the formation of colony forming units (CFUs) [240]. This neutrophilic response is inhibited in 5/6-nephrectomized mice as well as in mice treated with increasing FGF23 concentrations, whereas FGF23 blockade in 5/6-nephrectomized rats improves infiltration of neutrophils as well as reduces the formation of CFU in bronchoalveolar lavage (BAL) and spleen [240]. Fgfr2 knockdown by shRNA as well as application of pan-FGFR inhibitor, PD173074, restores neutrophil recruitment and reduces CFU in BAL, lung, and spleen [240]. Furthermore, FGF23 impairs neutrophil slow rolling induced by intercellular adhesion molecule 1 (ICAM-1), chemokine (C–X–C motive) ligand 1 (CXCL1) stimulated adhesion, and TNF-induced transmigration. However, functional assays show that Fgfr2 purely binds to αKlotho and does not bind to FGF23 [241,242]. Further studies are needed to resolve these contradictory data as well as establish the functions of FGF23 in different immune compartments.

In *Lepr^db/db^* mice, a mouse model of diabetic nephropathy, the expression of profibrotic genes and proteins as well as serum levels and renal expression of the inflammatory cytokines Il-6, Tnf, and Mcp-1 are reduced by injection of C-tail FGF23 peptide, which prevents iFGF23 signalling [243]. C-tail FGF23 does not affect serum iFGF23 and Pi levels [243]. If this is a direct effect of FGF23 signalling on both fibrosis and inflammation or indirect by reducing either of them, which leads to a decrease in fibrosis or inflammation, has to be determined.

Administration of LPS to wild-type mice increases Fgf23 gene and protein expression as early as the hepatic expression of the inflammatory cytokines *Tnf, Il6*, and *Il-1b* and causes hypoferremia as an acute phase response to infection and inflammation aiming to reduce iron availability for pathogens [244]. Administration of C-tail FGF23 peptide significantly reduces hepatic *Tnf* mRNA expression as well as hepatic and circulating hepcidin levels, which mitigated the LPS-induced hypoferremia [244]. Taken together, there is evidence of FGF23 signalling in response to inflammatory stimuli, but the exact function of FGF23 in the immune system remains elusive.

### FGF23 and calcitriol in immune cells

FGF23 and calcitriol are counterplayers in the regulation of mineral metabolism, but their mutual interaction in inflammation is not well understood. Human peripheral blood mononuclear cells (PBMCs) express αKlotho and FGFR 1, 2, and 4 receptors [245]. Calcitriol has a well-known modulatory function in the immune system and is involved in the antimicrobial defence and regulation of naïve T-cell development [246,247]. Activation of TLR1/2 and subsequent increase in IL-15 and IFNγ in the innate immune response triggers local calcitriol production in monocytes and macrophages by up-regulation of *Cyp27b1* mRNA expression [246,248]. Local increase of calcitriol levels allows the activation of the vitamin D receptor (VDR) in an auto- or paracrine way and the consequent up-regulation of cathelicidin, a protein with antimicrobial activity [246]. Low levels of calcidiol or blockade of 1-α-hydroxylase or VDR reduces the innate immune response [246]. In PBMCs, IL-15 treatment increases *Cyp27b1* mRNA expression with a concomitant increase in calcitriol production [245]. FGF23 inhibits IL-15-dependent stimulation of *Cyp27b1* expression and calcitriol production in PBMCs [245]. Furthermore, decreased calcitriol production induced by FGF23 is accompanied by down-regulation of calcitriol target genes, *Cyp24a1* and cathelicidin [245]. This suppressive effect of FGF23 on *Cyp24a1* in PBMCs is different from the kidney where FGF23 stimulates *Cyp24a1* expression. In PBMCs, FGF23 activates MAPK and AKT signalling, which is blocked by pre-treatment with the pan-FGFR inhibitor, PD173074 [245]. Whether these pathways mediate the effects of FGF23 to block IL-15 actions in PBMCs is unknown.

*Fgfr1*-expressing murine resident peritoneal macrophages increase *Tnf*, but not *Il-1b* and *Il-6* mRNA and protein expression in response to high dose of FGF23 [228]. Furthermore, FGF23 increases macrophage proliferation, which is prevented by pre-treatment with the Fgfr1 inhibitor, SU5402 [228]. The murine macrophage cell line, RAW264.7 expresses Fgfr1 and low levels of αKlotho in non-polarized M0 and anti-inflammatory M2 macrophages. However, αKlotho expression in pro-inflammatory M1 macrophages is markedly increased [249]. RAW264.7 treated with FGF23 activate ERK1/2 and increase TNF expression, which is prevented by pre-treatment with the FGFR inhibitor PD17307 as well as the ERK1/2 inhibitor U0126 [249]. Calcitriol prevents FGF23-induced up-regulation of *Tnf* mRNA expression in M0 macrophages, but not in pro-inflammatory M1 macrophages. In contrast, FGF23 has no effect on the anti-inflammatory arginase 1 (*Arg1*) mRNA expression and does not prevent calcitriol-induced up-regulation of *Arg1*. However, FGF23 prevents IL-4-induced Arg1 expression in M2 macrophages. Interestingly, FGF23 in combination with calcitriol up-regulates *Cyp24a1* expression in M0, M1, and M2 macrophages, but has no effect by itself. In contrast, FGF23 treatment increases *Cyp27b1* expression in M0 and M2, but not in M1 macrophages. Taken together, FGF23 inhibits the anti-inflammatory response of human and mouse macrophages to pathogenic stimuli, in part by regulation of calcitriol production, however, this has to be proven *in vivo*.

## Therapies to prevent or reduce vascular calcification

Based on the mechanisms described above, several lines of therapies have been developed or are currently in various stages of preclinical and clinical testing. This section will briefly mention some of these therapies.

### Pi-lowering therapies

Various types of Pi-lowering therapies are currently in use or in testing. These therapies include dietary Pi restriction, the use of Pi-binders, and more recently also drugs blocking intestinal absorption of Pi. Also, substances that block renal Pi reabsorption have been developed, but obviously depend on sufficient renal function [250]. Pi-restricting diets are effective in lowering blood Pi levels and associated biochemical markers, but their implementation is often difficult when trying to avoid too strong protein restriction in patients with advanced CKD. Pi binders may provide at best modest reductions in serum Pi and FGF23 levels and a recent well-controlled clinical trial assessing vascular stiffness through pulse-wave velocity as primary outcome failed to demonstrate efficacy, while bill burden and costs for this therapy are high [73,251]. More recently, tenapanor, a substance reducing paracellular intestinal Pi absorption, has been shown to reduce plasma Pi levels, but its effects on relevant clinical outcomes have not been tested to date [250,252,253].

### Prevention of calcium–Pi precipitation

Various strategies emerged to inhibit calcium–Pi crystallization. Lowering pH reduces vascular calcification [254], but the clinical relevance is questionable due to other detrimental effects of acidosis [255]. Sodium thiosulfate has been discussed to enable calcium exchange from insoluble deposits and may exert vasodilatory and antioxidant effects, but may also induce acidosis [256,257]. However, the risk of acidosis was reduced by sodium thiosulfate administration during dialysis with regular bicarbonate controls. And although sodium thiosulfate administration failed to reduce progression of abdominal aorta calcification, calcification progression in iliac arteries and cardiac valves was reduced [257]. SNF472 is an intravenous formulation of myo-inositol hexaphosphate, which blocks hydroxyapatite growth [258]. In a placebo-controlled study, SNF472-treatment of hemodialysis patients reduced the progression of coronary artery and aortic valve calcification [259]. Another concept is the treatment with vitamin K, which is required for activation of the calcification inhibitor matrix-GLA protein [260]. However, in a first clinical study, improvement of vitamin K status in dialysis patients did not prevent progression of vascular calcification [261]. In addition, magnesium interferes with CPP maturation [262] and prevents VSMC calcification [263,264]. In animal experiments, magnesium treatment reduced vascular calcification, but induced osteomalacia [265]. In hemodialysis patients, higher dialysate magnesium concentrations reduced serum calcification propensity [266]. Magnesium oxide treatment reduced progression of coronary artery calcification in CKD patients [267]. The impact of these strategies on cardiovascular outcomes requires further study.

### Calcimimetics

Calcimimetics activate the CaSR to reduce PTH secretion and prevent secondary hyperparathyroidism. Intriguingly, at least in parathyroid cells, Pi inhibits CaSR activity [268]. However, the CaSR is also expressed in many other organs and cells including vasculature [269]. Thus, calcimimetics may act also directly on vasculature and modulation of CaSR expression/activity has been associated with vascular calcification *in vitro* [270,271]. Stimulation of CaSR activity is expected from these models to reduce vascular calcification. The effect of cinacalcet, a first generation calcimimetic on vascular calcification or cardiovascular mortality was tested in the ADVANCE and EVOLVE trials. The ADVANCE trial showed a blunted increase of Agatston coronary artery calcification scores with cinacalcet treatment, which did not reach statistical significance. The EVOLVE study had no difference in the unadjusted primary outcome, but after adjustment an improved outcome was detected in the treatment arm. However, the conclusions for the EVOLVE trial are weakened by high drop-out rates, a poor definition of secondary hyperparathyroidism levels and the use of vitamin D [272,273].

### Interference with pro-inflammatory signalling pathways

Inhibition of inflammatory responses has emerged as putative strategy to ameliorate the effects of Pi on vascular calcification. Inhibition of TNF by neutralizing antibody reduces vascular calcification in a CKD mouse model [119]. Moreover, impaired NF-kB activation by SGK1 inhibition is sufficient to ameliorate vascular calcification [109]. Zinc supplementation is able to blunt activation of NF-kB and osteo-/chondrogenic transdifferentiation in VSMCs as well as vascular calcification during high Pi conditions, via G protein-coupled receptor 39 (GPR39)-mediated induction of TNF α-induced protein 3 (TNFAIP3) [274]. Zinc treatment further abrogates the pro-calcific effects of HIF prolyl hydroxylase inhibitors on VSMCs [275]. A similar anti-calcific effect of zinc was observed in valvular interstitial cells [276]. In addition, zinc may directly improve serum calcification propensity [277]. Serum uromodulin, a protein expressed in the kidney and released into circulation, may play a role as endogenous modulator of VSMC calcification [278]. Circulating uromodulin is reduced in CKD patients [278] and lower serum uromodulin concentrations are associated with mortality and cardiovascular events in CKD patients [279]. Uromodulin ameliorates oxidative stress by blocking transient receptor potential cation channel, subfamily M, member 2 (TRPM2), and circulating uromodulin levels correlate with markers of systemic oxidative DNA damage in AKI patients [280]. Uromodulin may function as a cytokine trap [281] and is able to ameliorate pro-calcific effects of TNF and IL-1β on NF-kB activation in VSMCs [282]. However, the effects of uromodulin on inflammatory processes are complex, and uromodulin can induce pro-inflammatory effects [283]. Furthermore, uromodulin overexpression failed to prevent calcification in a CKD mouse model, an effect presumably attributed to inhibition of uromodulin function by carbamylation [282]. Further research is required to elucidate the complex immunomodulatory role of uromodulin and its role in the link between CKD and a pro-calcific status of the vasculature. Another factor associated with pro-inflammatory effects in CKD is aldosterone [284]. Aldosterone promotes VSMC calcification [155], while treatment with the mineralocorticoid receptor antagonist spironolactone is able to ameliorate calcification in animal models [285,286] and explanted aortic rings [287]. Patients with primary aldosteronism exhibit increased abdominal aortic calcifications [288]. In CKD, hyperkalaemia following aldosterone antagonism is a key concern, but at least in dialysis patients, spironolactone treatment appeared safe [289,290]. Beneficial cardiovascular effects of aldosterone-antagonism were suggested [291], and clinical outcome trials are ongoing, which may pave the way for a more routine use of spironolactone in dialysis patients [292].

### Vitamin D

Vitamin D has been considered a “double edged sword” in vascular calcification [293]. Both, pro- and anti-calcific effects of active vitamin D were described in VSMCs [271,294]. Human arteries express components of the vitamin D system [295] and extra-renal formation of calcitriol may be relevant for vascular calcification in CKD [271,294,296]. Vitamin D has been tested in various clinical trials to prevent or reduce vascular calcification and cardiac complications in patients, particularly patients with CKD, based on data from rodent models showing beneficial effects on the heart and vasculature, the association of low vitamin D levels and CVD in epidemiological studies, and the ability of calcitriol to reduce the activity of the RAAS and to suppress PTH secretion [293]. Also, patients with CKD suffer frequently from low calcitriol levels. The randomized, placebo-controlled VITAL trial in men older than 50 years and women older than 55 years did not find any evidence for a reduction in cardiovascular end-points in vitamin D-supplemented participants [297]. Likewise, a series of other smaller and larger studies (e.g. the PRIMO and OPERA trials) found conflicting data on effects of vitamin-D supplementation on blood pressure and cardiac parameters. Thus, evidence from well-controlled interventional trials with sufficient power are missing to demonstrate relevant changes in cardiovascular outcomes [272].

## Summary and outlook

CVD is a major cause of morbidity and mortality in industrialized countries. Amounting evidence hints at a complex interaction between Pi and its hormonal regulators and inflammation. The cardiovascular alterations associated with these pro-inflammatory effects could link Pi homeostasis to CVD and mortality not only in very vulnerable patients with CKD, but also in the general population with intact kidney function. Despite being a multifactorial disease, vascular calcification may be an important contributor to the cardiovascular effects of Pi. Although first therapeutic concepts are emerging, many gaps remain in the understanding of the complex interplay orchestrating the systemic and cardiovascular response to mineral stress by local and/or systemic Pi disbalance.

## Abbreviations

AKI: acute kidney injury
*Anp*: atrial natriuretic peptide
BAL: bronchoalveolar lavage
*Bnp*: brain natriuretic peptide
CaSR: calcium-sensing receptor
CBFA1: core-binding factor α1
CFU: colony forming unit
CKD: chronic kidney disease
CNT: connecting tubule
CPP: calciprotein particle
CRIC: Chronic Renal Insufficiency Cohort
CRP: C-reactive protein
CVD: cardiovascular disease
CXCL1: CXC chemokine ligand
DCT: distal convoluted tubule
ENPP: ectonucleotide pyrophosphatase/phosphodiesterase
ESRD: end-stage renal disease
FA-AKI: folic acid-induced AKI
FGF23: fibroblast growth factor 23
hs-CRP: high sensitivity CRP
IBD: inflammatory bowel disease
IGF-1: insulin-like growth factor 1
IKK: inhibitor of nuclear factor κ B (IkB) kinase
IL: interleukin
KC: keratinocyte chemoattractant
LPS: lipopolysaccharide
LVH: left ventricular hypertrophy
MAPK: mitogen-activated protein kinase
*Mcad*: medium chain acyl-CoA dehydrogenase
MCP-1: monocyte chemoattractant protein 1
*Myh7*: β-myosin heavy chain
NFAT: nuclear factor of activated T cells
NF-kB: nuclear factor κ-light-chain-enhancer of activated B cells
NRF2: nuclear factor erythroid 2-related factor 2
NRVC: neonatal rat ventricular cardiomyocytes
NT-pro-BNP: N-terminal pro-brain natriuretic peptide
PBMC: peripheral blood mononuclear cell
Pi: inorganic phosphate
PLCγ: phospholipase C γ
PPi: pyrophosphate
PTH: parathyroid hormone
RAAS: renin–angiotensin–aldosterone system
TGFβ: transforming growth factor β
TLR: Toll-like receptor
TNF: tumor necrosis factor
VDR: vitamin D receptor
VSMC: vascular smooth muscle cell
XLH: X-linked hypophosphatemic rickets
